# Disseminated Anaplastic Lymphoma Kinase (ALK)-Positive T-cell Lymphoma Involving the Uterus and Cervix: A Case Report

**DOI:** 10.7759/cureus.52815

**Published:** 2024-01-23

**Authors:** Erin J Yang, Ahmed Sabri, Seifeldin Awad, Lesley B Conrad, David Cantu

**Affiliations:** 1 Department of Obstetrics and Gynecology, Creighton University School of Medicine, Omaha, USA; 2 Department of Pathology, Creighton University School of Medicine, Omaha, USA

**Keywords:** gynecology, immunohistochemistry, adult t-cell lymphoma, abnormal uterine bleeding, rare cancers, rare metastases, alk-positive, disseminated disease, t-cell lymphoma, non-hodgins lymphoma

## Abstract

Primary or secondary non-Hodgkin lymphomas (NHLs) involving the female gynecologic tract are rare. T-cell subtypes are further rare and portend a worse prognosis. We present a case of a 23-year-old female presenting with a cervical mass accompanied by constitutional symptoms and abnormal vaginal bleeding. Immunohistochemistry studies revealed the presence of disseminated T-cell non-Hodgkin lymphoma that was anaplastic lymphoma kinase (ALK)-positive. The patient demonstrated a complete response to systemic chemotherapy initially and again after the relapse of the disease one year after diagnosis. To our knowledge, this is the first case of an ALK-positive T-cell lymphoma with secondary involvement of the uterus and cervix; all previously published cases of this histologic subtype in the gynecologic tract describe primary disease of the vagina. This case emphasizes the importance of immunohistochemistry studies inclusive of T-cell and B-cell markers when evaluating biopsies from cervical tumors to render the appropriate diagnosis and guide systemic therapy.

## Introduction

Non-Hodgkin lymphoma (NHL) of the female gynecologic tract, either primary manifestation or secondary involvement from disseminated disease, is rare [1]. The most commonly affected area are the ovaries, and the most common complaint prompting consultation is abnormal vaginal bleeding [1,2]. The majority of cases are of B-cell origin, with diffuse large B-cell lymphoma (DLBCL) being the most common histologic subtype [1,3]. T-cell lymphomas involving the gynecologic tract are far less common and typically have a poor prognosis [1-7]. Herein, we describe a case of anaplastic lymphoma kinase (ALK)-positive anaplastic large cell lymphoma (ALK+ ALCL) with secondary involvement of the uterus and cervix successfully treated with systemic chemotherapy. Only two cases of the same histologic subtype involving the gynecologic tract have been reported to date, both of which describe primary disease of the vagina [5,6].

## Case presentation

**Figure 1:**
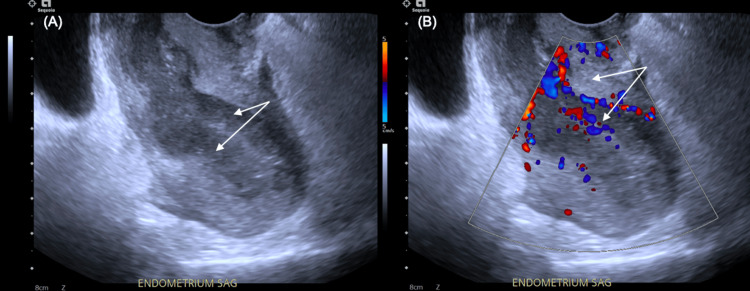
Transvaginal ultrasound, sagittal view of the uterus. (A) Transvaginal ultrasound demonstrates an 8-cm retroflexed uterus with increased thickness of the sub-endometrial hypoechoic halo (arrows). (B) Color Doppler demonstrates areas of increased vascularity and small cysts within the endometrium (arrows).

A 23-year-old Caucasian female with a past medical history of hypothyroidism, obesity (body mass index (BMI): 32 kg/m^2^), and polycystic ovarian syndrome presented to an out-of-state emergency department due to consistent vaginal bleeding for eight consecutive days and hemoglobin of 7.2 gm/dL. One unit of packed red blood cells was administered. She was discharged home on oral medroxyprogesterone 10 mg daily and recommended to follow up with a gynecologist. She then re-presented to a local emergency room two days later with continued menorrhagia. Pelvic and transvaginal ultrasound demonstrated an 8-cm retroflexed uterus with increased thickness of the sub-endometrial hypoechoic halo, as well as areas of increased vascularity and small cysts within the endometrium concerning for adenomyosis (Figure 1A, 1B). Complete blood count demonstrated leukocytosis (20.7 k/uL) (absolute neutrophils: 16.1 k/uL), thrombocytosis (672 k/uL), and mild anemia (hemoglobin: 10.6 gm/dL, hematocrit: 34.9%). Vital signs were significant for mild tachycardia. She was discharged and instructed to increase oral medroxyprogesterone to 20 mg daily and follow up with a gynecologist.

**Figure 2:**
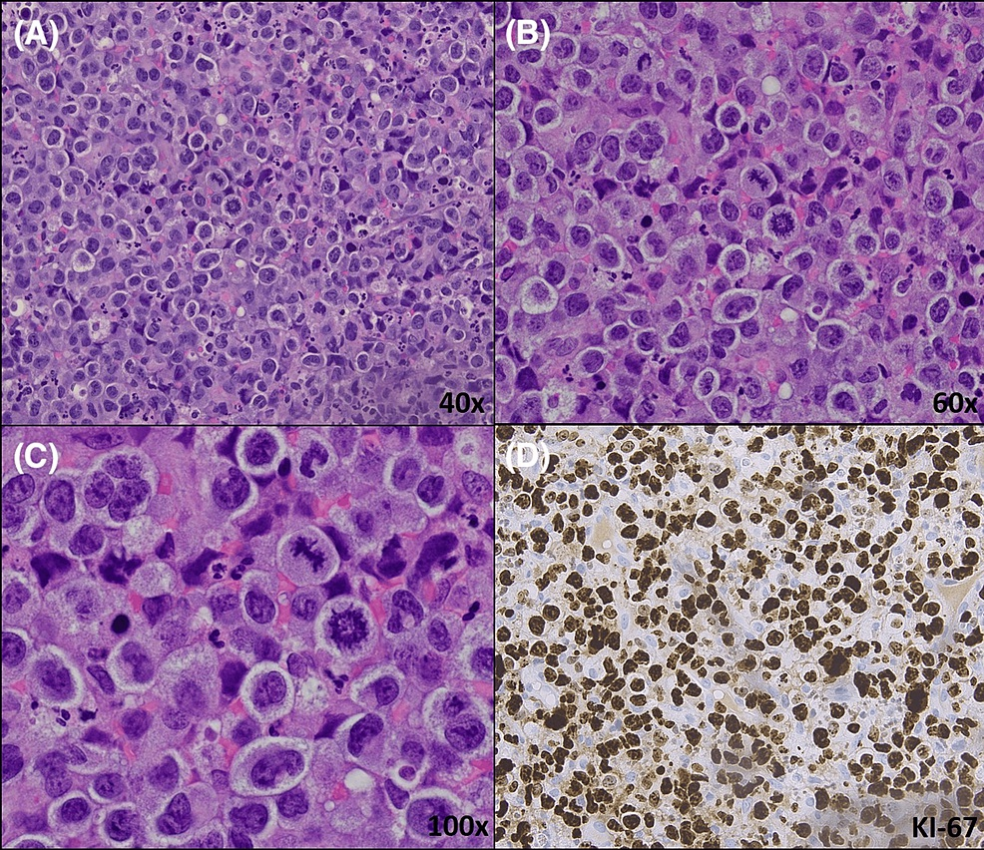
ALK-positive anaplastic large cell lymphoma on H&E stain (40×, 60×, and 100×) and Ki-67 stain. (A-C) Sheets of large pleomorphic cells and “hallmark cells” are present on H&E stain. These cells have irregular nuclear borders with variable nuclei and inconspicuous nucleoli, along with an abundant eosinophilic cytoplasm. In addition, there is extensive mitotic activity and apoptotic bodies in a background of acute inflammation. (D) Ki-67 stain shows a high proliferation index. H&E: hematoxylin and eosin

**Figure 3:**
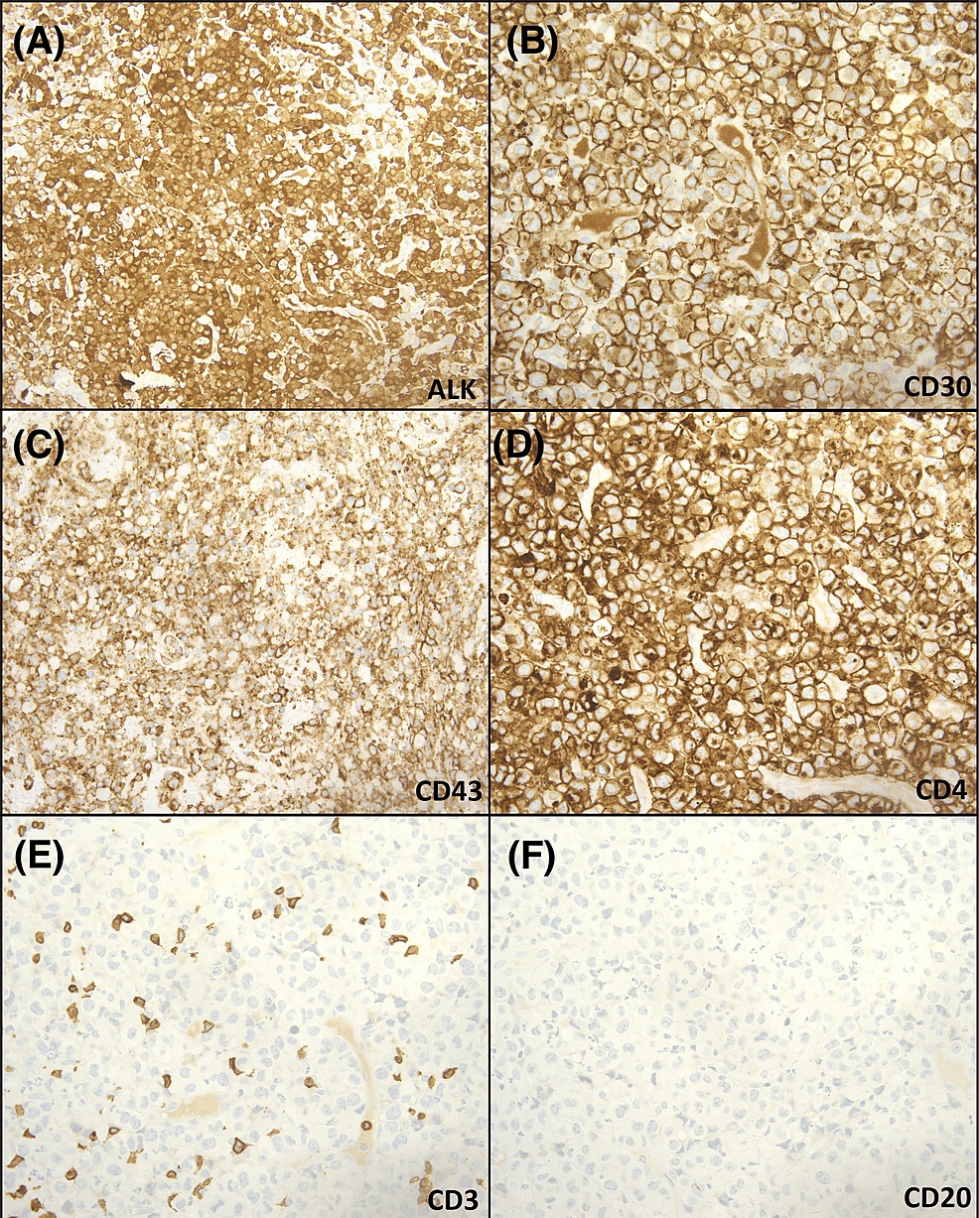
Immunohistochemical stains (40×). The malignant cells show nuclear and diffuse cytoplasmic staining for (A) ALK, membranous and Golgi type staining for (B) CD30, membranous staining for (C) CD43, and cytoplasmic with membranous accentuation staining for (D) CD4. The malignant cells are negative for (E) CD3 and (F) CD20. ALK: anaplastic lymphoma kinase, CD: cluster of differentiation

On presentation to her gynecologist three days later, a speculum examination revealed a cervical lesion present at six o’clock with active bleeding. A biopsy was obtained and demonstrated sheets of large pleomorphic cells with extensive mitotic activity and apoptotic bodies in a background of necrosis, hemorrhage, and acute inflammation. These cells were of lymphoid lineage and had irregular nuclear borders with variably centered to eccentric nuclei and inconspicuous nucleoli, along with abundant eosinophilic cytoplasm. The characteristic hallmark cells were also present (Figure 2A-2D). By immunohistochemistry, the malignant cells were strongly positive for ALK, CD30 (TNF receptor superfamily member 8), CD10 (neutral endopeptidase), cluster of differentiation 4 (CD4), and CD43 (leukosialin or sialophorin) (Figure 3A-3F). LCA (leukocyte common antigen) (CD45) showed membranous positivity in the majority of cells. B-cell lymphoma 6 (BCL-6) showed weak nuclear positivity in a subset of cells. Granzyme B was positive in a subset of malignant cells with a granular staining pattern. ALK staining showed nuclear and diffuse cytoplasmic positivity in the malignant cells. The Ki-67 proliferation index was increased. Cluster of differentiation 15 (CD15) was positive in a subset of cells. The malignant cells were negative for cytokeratin stains (anion exchanger 1 (AE1)/anion exchanger 3 (AE3), cytokeratin 7 (CK7), and cytokeratin 20 (CK20)), Sry-related HMG-BOX gene 10 (SOX-10), Melan A, CD20, CD3, CD2, CD5, BCL-2, paired-box gene 8 (PAX-8), PAX-5, T-cell intracellular antigen 1 (TIA-1), CD79a (B-cell antigen receptor complex-associated protein alpha chain), and Epstein-Barr encoding region in situ hybridization (EBER-ISH). Fluorescence in situ hybridization (FISH) was positive for rearrangement of the ALK (2p23) locus. The constellation of these findings is consistent with ALK+ ALCL. A bone marrow biopsy along with ancillary studies were performed later and were unremarkable.

**Figure 4:**
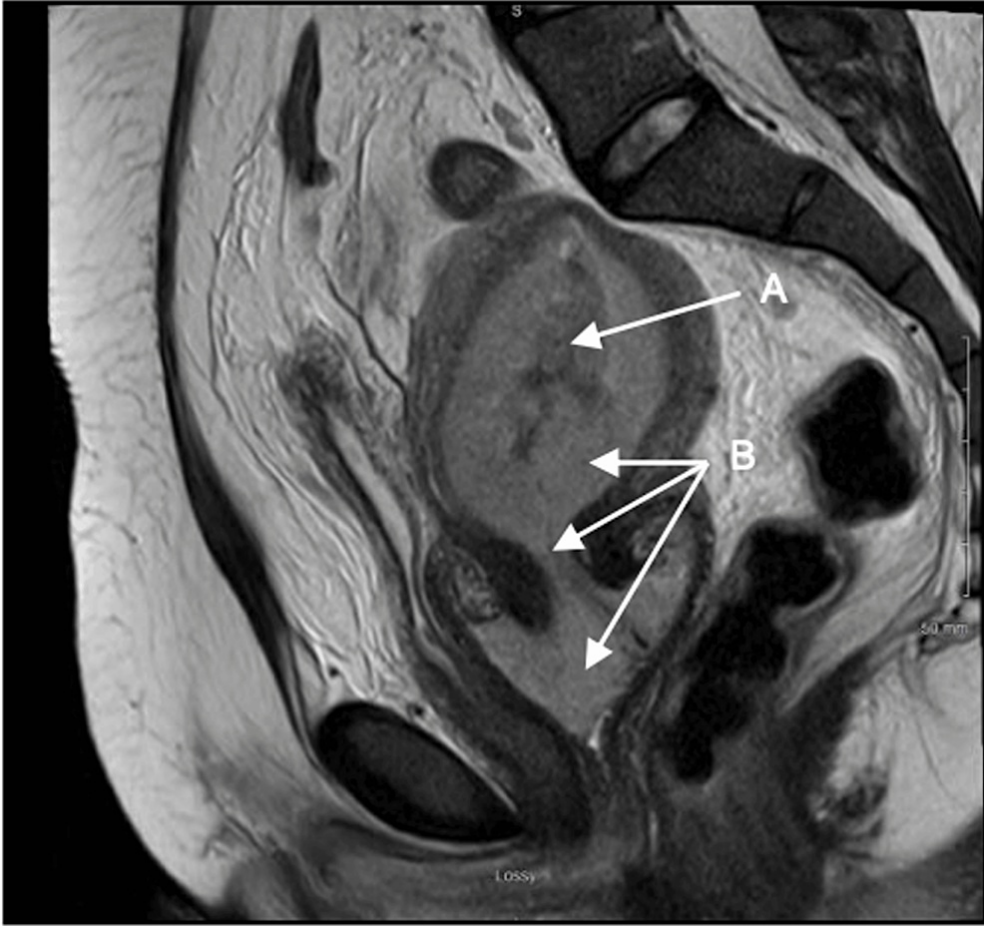
MRI of the pelvis, sagittal view. MRI of the pelvis demonstrates a (A, arrow) heterogeneous T2 hypointense region in the endometrium measuring 2.2 cm in thickness with areas of T2 hypointensity. (B, arrow) Intermediate T2 hyperintense sub-endometrial soft tissue surrounding the endometrial stripe, extending into the endocervical canal, and distending the vaginal cavity. MRI: magnetic resonance imaging

Metastatic workup included PET/CT, MRI pelvis, lactate dehydrogenase (LDH), and bone marrow biopsy (Figure 4). PET/CT demonstrated nodal lesions above and below the diaphragm, and several extranodal sites, including pulmonary, mediastinal, liver, uterine, cervix, and T10 vertebra with an associated compression fracture. LDH was elevated at 323 U/L. The bone marrow biopsy showed normocellular bone marrow, and chromosome analysis demonstrated a normal female chromosome complement with no cytogenetic evidence of an abnormal clone. Final staging was consistent with Ann Arbor stage IV disease and an International Prognostic Index (IPI) score of 3 (one point stage, one point baseline LDH, one point extranodal disease) with an estimated four-year progression-free and overall survival of 53% and 55%, respectively.

She subsequently presented to the emergency department with severe lower back pain, nausea, vomiting, and anorexia. She was admitted for intravenous hydration and pain control. She received intrathecal methotrexate for central nervous system (CNS) prophylaxis followed by one cycle of cyclophosphamide, doxorubicin, vincristine, and prednisone (CHOP) while inpatient. To further guide systemic therapy, the CD30 cell positive percentage was assessed by immunohistochemistry and found to be greater than 10%. Thus, systemic chemotherapy with cyclophosphamide, doxorubicin, prednisone, and the addition of brentuximab vedotin (BV-CHP) was administered for cycles two through six. She was readmitted between cycles two and three for bilateral pulmonary emboli and initiated on rivaroxaban. Restaging PET/CT after cycle 4 demonstrated complete resolution of the disease. Two consolidation cycles were given, and she underwent T10 kyphoplasty to relieve pain associated with the prior metastatic lesion and compression fracture at this site. Post-treatment PET/CT demonstrated no lesions. Surveillance scans five months later (approximately one year after diagnosis) demonstrated mediastinal and hilar lymphadenopathy as well as a 3 cm left breast mass. The breast mass was biopsied and consistent with a relapse of her lymphoma. She received four cycles of gemcitabine, dexamethasone, and cisplatin therapy (GDP) with a complete response to treatment on imaging. She then completed seven more cycles of GDP and was evaluated for a bone marrow transplant. PET/CT imaging upon treatment completion demonstrated a complete response. Two months later, the patient underwent an allogeneic stem cell transplant. She did develop acute graft versus host disease, which is currently managed with tacrolimus.

## Discussion

The morphological differential diagnosis in our case includes poorly differentiated carcinoma, melanoma, sarcoma, lymphoma, and others. Immunohistochemistry studies demonstrated a lymphoid origin with positive CD30, CD10, CD4, and CD43, and LCA (CD45) with negative melanoma markers such as SOX-10 and Melan A, and negative carcinoma markers such as CK AE1/AE3, CK7, and CK20. Additionally, a diagnosis of sarcoma and soft tissue tumors is less favored due to morphology CD30 positivity. Other lymphomas or atypical lymphoid diseases are ruled out based on morphology and immunohistochemistry, such as Hodgkin lymphoma or even to a lesser extent lymphomatoid granulomatosis. The differential diagnosis at this point includes ALK-positive large B-cell lymphoma (ALK+ LBCL) and ALK-positive anaplastic large cell lymphoma (ALK+ ALCL). Both lymphomas can express CD30 and have similar morphology. Therefore, T-cell and B-cell markers are extremely important to differentiate between these two entities [7]. In our case, a diagnosis of ALK+ ALCL was rendered due to the lack of B-cell markers and the expression of a subset of T-cell markers.

To our knowledge, this is the first report of ALK+ ALCL with dissemination to the gynecologic tract. Only two reports of the same histologic subtype involving the gynecologic tract appear in the literature, both of which describe primary extranodal ALK+ ALCL of the vagina [5,6]. The first report describes stage II disease with involvement of the bladder that initially presented as a vaginal mass in a 52-year-old with chronic renal failure. Immunohistochemically, this case shared positive CD30 and ALK markers with our case; however, positive CD3 and negative CD43 were also present, which were the opposite findings in our case. Unfortunately, the patient deteriorated quickly and died two weeks after the initial biopsy before treatment could be initiated [5,6]. The second report describes stage I disease that initially presented as a vaginal lesion with associated fevers in a healthy 35-year-old. Immunohistochemistry was positive for CD4, CD43, CD30, and ALK, identical to our case. The patient had a complete response to treatment with systemic chemotherapy (BV-CHP) as of one-year follow-up [6]. The patient’s outcome beyond this time is unknown. Our patient similarly had a complete response to BV-CHP but did relapse approximately one year after diagnosis.

All subtypes of T-cell lymphoma involving the gynecologic tract are exceedingly rare. Thus, the optimal treatment protocol for these tumors is unclear. Most treatments involve surgical resection with simple hysterectomy and bilateral salpingo-oophorectomy, chemotherapy, irradiation, and their combinations [7]. In reviewing the literature, we identified four reports describing successful treatment of primary T-cell lymphoma, including (1) stage I precursor T-lymphoblastic lymphoma of the ovary treated with surgical resection and chemotherapy, (2) stage I NK/T-cell lymphoma of the vagina treated with surgical resection, (3) stage I peripheral T-cell lymphoma of the vagina treated with chemotherapy (THP-COP) and radiotherapy, and (4) stage I peripheral T-cell lymphoma of the uterus treated with surgical resection and chemotherapy (CHOP) [1,4]. We also found two reports describing the treatment of secondary T-cell lymphoma, including (1) stage IV peripheral T-cell lymphoma involving the vulva treated with chemotherapy, in which the patient died five years after diagnosis, and (2) stage IV cutaneous T-cell lymphoma (mycosis fungoides) involving the vulva treated with chemotherapy and phototherapy, where the patient was alive as of six years after initial diagnosis [1]. Of these existing reports, none describe the ALK-positive subtype or recurrent disease as in the case of our patient [1,4].

## Conclusions

In conclusion, we have described what we believe is the first report of secondary ALK+ ALCL involving the gynecologic tract successfully treated with targeted chemotherapy. Relapse occurred approximately one year after diagnosis, and the patient was again successfully treated with chemotherapy followed by allogeneic bone marrow transplant. This case emphasizes the importance of immunohistochemistry studies to differentiate this lymphoma from other lymphoma types and from other malignancies that could mimic lymphoma. Given the rare incidence of NHL involving the gynecologic tract, particularly the ALK+ and T-cell subtypes, this report hopes to guide diagnosis and treatment for future cases.
